# Memoir on Glaucoma

**Published:** 1843-07

**Authors:** 


### Art. VI.

*Mémoire sur le Glaucôme.* Par le Docteur Jules Sichel. *Extrait des Annales d'Oculistique*, Vols. V. VI. et VII. — *Bruxelles,* 1842. 8vo, pp. 260.

*Memoir on Glaucoma.* By Dr. J. Sichel.—*Brussels,* 1842.

In this work, Dr. Sichel claims our attention in the triple character of a critic, a pathologist, and a medical historian ; and notwithstanding certain serious offences which he has committed, especially in the execution of the last of these offices, we have no hesitation in awarding him the praise due to labour and perseverence in his researches, and to the merit of having produced a book which will not merely repay the perusal of those interested in the pathology of the eye, but which, we conceive, they are bound to study with the greatest care, as containing by far the most complete account of a very serious disease of the organ of vision.

The scope afforded, on the present occasion, to Dr. Sichel's critical powers, arises from the obscurity attached to the meaning of the Greek word γλαυκὸς. The use made by Homer of the same word, to designate the blue colour of the eyes of Minerva, γλαυκῶπις 'Αθήνη, and the blueish-green colour of the sea, γλαυκὴ θάλασσα, is sufficient to show the original ambiguity of the term. As if, however, on purpose to puzzle the critics, Virgil applies the word to the colour even of horses, *honesti spadices glaucique*. Dr. Sichel thinks, that by *glauci* Virgil meant gray, for which interpretation he assigns, with all due gravity, the very satisfactory reason, that no one ever saw a green horse—" puisqu' aucun individu de l'espèce caballine ne présente dans sa robe le moindre mélange d'une nuance tirant sur le vert."

Dr. Sichel has satisfied us, that the word γλαυκὸς was applied by the ancient Greeks to the blueish or grayish colour of the iris, presented by a large portion of the human race, and that it was thence transferred to certain states of the eye, in which the pupil has no longer the natural jet black colour of youth and health, but has assumed the grayish or greenish tinge of age and disease. Some will have it that Hippocrates, under the plural γλαυκώσεες, comprehended all those diseases of the eye in which an *opaque* appearance is seen within the pupil;* but Dr. Sichel supposes that he refers to cataract only, and that he had no notion of the *glaucoma* of modern oculists.

The pupil of a young and healthy eye presents a jet black colour, and one unacquainted with the real state of matters might suppose, on looking into such a pupil, that the whole of the light which entered it passed unobstructed to the bottom of the eye, and was there absorbed by the choroid pigment. This, however, is not the case; but, as was first shown by Purkinje,† a quantity of the light is arrested by the anterior crystalline capsule, and another quantity by the posterior, both quantities being reflected back again through the pupil and out of the eye. If the source of the light so reflected be a small luminous object, such as a lighted candle, two distinct images are formed of the object, the one erect and the other inverted, as is readily perceived by the spectator. This catoptrical effect of the two membranes forming the capsule of the lens, since it was first announced by Purkinje, has been studied with considerable attention by Sanson and others,‡ as a means of detecting various diseased states of the crystalline body; and although its value seems very insufficiently known to Dr. Sichel, we look upon it not only as one of the most ingenious and beautiful applications of optical principles to the illustration of disease in the eye, but also as one of the most sure and useful. A certain size, brightness, and colour attend the images in the young and healthy eye, but are modified by whatever disease affects the lens or its capsule. Among the diseases which modify the images is glaucoma.§

If we look into the eye of an old person, we very rarely find the pupil of a jet black colour. On the contrary, it presents more or less of a

* Mackenzie, On Glaucoma. Glasgow Medical Journal for August, 1830, p. 254.

† Purkinje, De Examine physiologico Organi Visus, &c.—Vratislaviæ, 1823.

‡ Mackenzie, On Lenticular Glaucoma. London Medical Gazette, vol. xvi. p. 107.—London, 1838.

§ We need scarcely mention that the two images referred to in the text are independent of the image reflected by the cornea.

greenish hue, and this is what constitutes strictly and truly glaucoma. If we take a lighted candle, and move it in front of such an eye, we immediately discover, that, while both the erect image formed by the anterior capsule, and the inverted one formed by the posterior, are distinct, the erect one is larger and brighter than in the young and healthy eye, and has somewhat of a yellowish hue. The inverted image is also larger and more of a yellowish colour than in the young eye, and not unfrequently its outline has lost its original sharpness and become somewhat diffused. These changes indicate the *first* degree of glaucoma. They are attended by a lively pupil, and a distinct vision of distant objects: they are the result of the lens having assumed a yellow or amber colour, and of its kernel having acquired a greater density than what it possessed in youth.

What we have now stated is altogether at variance with the opinions of Dr. Sichel. He will not admit that the greenish appearance which we have just described has any claim to be considered as a glaucoma at all (p. 30), but insists that it is merely what he calls "un reflet particulier du cristallin sur la choroide." "A particular reflection of the crystalline on the choroid" does not appear to be a very accurate mode of expression. Perhaps what Dr. Sichel meant to say was this, that the light entering the eye, being refracted by the crystalline, is brought to a focus on the choroid, and is thence reflected through the humours of the eye, so as to be seen by a spectator; but we are convinced that the appearance which Dr. Sichel designates by the name of "reflet particulier du cristallin" is a reflection, not from the choroid, but from the crystalline itself, and is a result, along with the changes in the images already referred to, of the kernel of the lens having assumed an amber hue, and acquired a greater degree of firmness, and therefore a greater reflective power than what it possesses in the young and healthy eye. It is easy to ascertain the truth of this, by opening the cornea and extracting the lens from the eye after death, when the glaucomatous appearance will be seen to be entirely removed. If Dr. Sichel will only throw the light, refracted to a focus by a double convex lens, on his hand or on a sheet of paper, and try to see it reflected from the hand or the paper, through the lens, we think he will give up the notion of any similar reflection being seen from the choroid through the crystalline. Were there a window in the sclerotica, through which the doctor might peep without looking through the lens, then, indeed, something might be seen, resembling "le foyer d'une loupe mobile, qu'on promenerait au-dessus d'un fond opaque," (p. 30,) but to discern anything like this through the crystalline is impossible.

The glaucomatous reflection from the lens of an old person generally increases slowly, as age advances; but so long as it is combined with a lively pupil, distinct vision of distant objects, and a natural firmness of the eyeball to the touch, we should call it the *first* degree of glaucoma, or a simple uncomplicated glaucoma. In what may be styled the *second* degree, the greenish reflection is more striking, the eyeball assumes a preternatural degree of hardness, the motions of the pupil become limited and slow, and the vision of distant objects by the naked eye and of near objects through convex glasses is more or less disturbed. On examining the eye catoptrically, the inverted image is distinct when formed near the edge of the crystalline, but becomes less and less distinct, as, by moving the candle, it is brought towards the centre of the pupil. Photopsia, pain in and round the eye, partial changes in the colour of the iris, and a dilated state of the subconjunctival vessels, not unfrequently attend this stage.

In the *third* stage, the green appearance of the lens is still more remarkable, the eyeball becomes of a stony hardness, the pupil is dilated and fixed, and vision is extinguished. On examining the eye catoptrically, the inverted image is no longer visible, even at the edge of the lens. In this third stage, as also in the second, the erect image formed by the anterior capsule, is large and evident, but its outline is indistinct, so that it looks like a diffused blaze. The fact of its being so distinct, is to be attributed to the kernel of the lens having acquired, not merely an amber, but a reddish-brown colour, with a certain degree of opacity, so that it serves as a foil to the image.

A *fourth* stage may succeed, in which the lens becomes cataractous, as well as glaucomatous; and a *fifth*, in which the opaque lens, much hypertrophied, is pressed, through the pupil, against the cornea, till the cornea ulcerates and gives way, allowing the lens to escape, along with a considerable quantity of blood from the internal vessels of the eye.

While it may be a matter of doubt, whether Hippocrates employed the plural γλαυκώσεες to signify cataract only, or to comprehend every sort of opacity seen within the eye, it is certain that Rufus and Galen used the term γλαύκωμα for an opacity of the crystalline, and ὑπόχυμα to denote a concreted fluid occupying the posterior chamber. This concreted fluid, as they took it to be, (for it was in fact the lens in the state of cataract,) they found could be removed, and vision restored, by the operation of couching; glaucoma they declared to be incurable. The notion that cataract or ὑπόχυμα was a concreted fluid being refuted by Quarré, Lasnier, and others, in the early part of the 17th century, and the seat of that disease being ascertained to be the lens, the term glaucoma ran a chance of being lost, had it not been taken up by Brisseau, and applied by him to signify an opacity of the vitreous humour. This notion of Brisseau regarding the nature of glaucoma has, in a great measure, kept its ground down to this very hour, notwithstanding the light thrown on the pathology of the disease by the dissections of Dr. Mackenzie, an account of which he published in 1830, and from which the following are extracts:

"The following are the particulars which I observed.

"1. The choroid coat, and especially the portion of it in contact with the retina, of a light-brown colour, without any appearance of pigmentum nigrum.

"2. The vitreous humour in a fluid state; perfectly pellucid, colourless, or slightly yellow. No trace of hyaloid membrane.

"3. The lens of a yellow or amber colour, especially towards its centre; its consistence firm, and its transparency perfect, or nearly so.

"4. In the retina, no trace of limbus luteus, or foramen centrale.

"To the first of these changes, namely, the deficiency of pigmentum nigrum, I am inclined to ascribe, in a great measure, the opaque appearance of the deep-seated parts of the eye in glaucoma. This appearance I regard as a reflection merely of the light from the retina, choroid, and sclerotica; it is probably blueish when it first leaves the reflecting surface formed by these membranes, but immediately assumes a greenish hue from passing through the yellowish fluid which occupies the place of the vitreous humour, and through the lens, which is still more decidedly of a yellow, or even amber colour, at that period of life when glaucoma is most apt to attack the eye. . . . . . . .

"In my dissections of glaucomatous eyes, I have detected no other change in the retina than what I have already mentioned; namely, a want of the limbus luteus and foramen centrale. The membrane never appeared to be thickened or changed in colour; and as for the vitreous humour, instead of being thickened or opaque, as described by authors, it was always fluid and perfectly transparent. I by no means presume to assert that a turbid state of the vitreous fluid, or an opacity of the hyaloid membrane never occurs; nor do I deny that the retina, in certain cases, becomes thickened and opaque. What I believe myself warranted in maintaining is this, that the well-known appearances of glaucoma are independent of any of these changes, and to be attributed to certain other morbid alterations of the internal parts of the eye. . . . . . . .

"There is no green surface in the human eye to reflect the light of that colour, as there is in the eye of the sheep; it must be, then, in its transmission that it acquires the greenish hue, and the part most likely to affect it in this way is the lens. Were it proved that the retina, which is naturally somewhat blueish, supported by a choroid destitute of pigment and a whitish sclerotica, reflects the light forward into the eye of a blueish colour, then one of the principal phenomena of glaucoma might be regarded as no longer difficult of explanation. In confirmation of this, if the lens is removed in this disease, or sinks to the bottom of the dissolved vitreous humour, the green appearance is almost entirely lost."

We have been led to make these extracts from Dr. Mackenzie's first paper "On Glaucoma," published in the Glasgow Medical Journal for August 1830, because, strange to tell, Dr. Sichel, while with an extreme minuteness he refers to every other publication upon glaucoma, and while he makes frequent mention of Dr. Mackenzie's opinion on that disease, avoids all reference to the paper in question, as well as to the first edition of Dr. Mackenzie's Practical Treatise on the Diseases of the Eye, published also in 1830, and in which that paper was reprinted *verbatim*. The reason of this disingenuousness does not appear, till the very end of Dr. Sichel's work, where, in a summary of conclusions, he puts in the following claim to the very observations originally given to the world by Dr. Mackenzie in 1830:

"It was only in 1831, that M. Canstatt and myself made another important step in advance, as to the knowledge of the nature of glaucoma. We proved that the decoloration of the choroid, and the yellow hue of one of the refracting media, the vitreous body, and not the green opacity of the latter, are the true causes of the seeming greenish opacity of the bottom of the eye in this disease. In 1837, I showed that the crystalline, become yellowish, had more influence in producing this hue, than the vitreous body." (p. 258.)

On this occasion, then, Dr. Sichel has not acted "*en historien fidèle*;" what he states not to have been done till accomplished by himself and M. Canstatt in 1831 and in 1837, was already known to the profession in 1830; and we can assure him, that the leap which he makes in his chronological review, (p. 221,) from 1828, *Schoen*, to 1831, *Canstatt et Sichel*, however it may pass without detection in Paris or in Brussels, will at once appear to an English or a German reader in its true light.

It is plain, both from the symptoms during life, and the state of the eye after death, that various elements enter into the production of a glaucoma of the *third* degree, or what Dr. Sichel calls "*un vrai glaucome*." Pain and congestion are present, the one affecting the ramifications of the ophthalmic nerve, and the other the whole vascular structure of the eyeball. There is a change in the colour and consistence of the lens, the chief factor in the production of the greenish hue seen behind the pupil; an accumulation of dissolved vitreous humour, compressing the retina, and over-distending the tunics; an absorption of the choroid pigment; and an insensibility of the retina. These several elements are observed to coexist in different degrees, in different cases. In some, the retinal element precedes the rest, or is more than ordinarily advanced. The disease is then regarded as an amaurosis accompanied by glaucoma, and by some is called *amaurosis glaucomatosa*. In other cases, the lenticular element is the most remarkable; the hyaloid membrane and the retina are comparatively sound. The lens, having first of all grown yellow and glaucomatous, may now become cataractous; opaque, that is, on its surface, as well as glaucomatous in its kernel; and may be extracted with advantage. This constitutes the "*cataracte verte opérable*" of Dr. Sichel. If the glaucomatous lens becomes cataractous, after the hyaloid membrane is dissolved, the retina insensible, and the blood-vessels of the eye varicose, the disease is styled *cataracta glaucomatosa*, and is altogether hopeless.

Various views have been entertained as to the first and principal pathological change in glaucoma. Dr. Mackenzie seems to attribute the absorption of the choroid pigment, the insensibility of the retina, and even in part the glaucomatous change of the lens, to the pressure of a superabundant vitreous fluid. Mr. Tyrrell, who still holds to the exploded doctrine that the vitreous body is opaque, and that this is the cause of the glaucomatous appearance, considers the morbid action to be first set up in the retina, and thence to spread to the hyaloid membrane, and to the lens.* One of the peculiar doctrines of Dr. Sichel is, that inflammation of the choroid is the cause of glaucoma. This opinion he insists on with great earnestness, and we consider his reasonings on the subject as deserving serious consideration. If we admit with Dr. Sichel, that choroiditis is always the direct and principal cause of glaucoma, (p. 98,) it is somewhat remarkable that this effect follows only in those who are past middle life, and that inflammation of the choroid produces no such change in the transparent media of the eye in young persons. He tells us, that his dissections have always presented manifest signs of disorganization, more or less extensive, of the choroid; and we know that, in the *fourth* stage, the choroid and retina have occasionally been so much changed as scarcely to be recognized. We must confess, however, that the signs of disorganization mentioned by Dr. Sichel, namely, the injection of the vasa vorticosa by blood, the thinness of the choroid, and the deficiency of pigment, scarcely seem sufficient for the foundation of so positive and unrestricted an opinion on the choroidal origin of the disease, as that which prevades the work before us.

Dr. Sichel announces (p. 1,) that his special object is the pathological anatomy of glaucoma, hitherto, he says, very incompletely studied. But, as far as we have discovered, he has made no remarkable addition, under this head, to what was previously known. He seems anxious to make out the choroid to be of a blue colour, (p. 87,) in order to explain the

* Tyrrell, Practical work on the Diseases of the Eye, vol. ii. p. 134.—London, 1840.

green hue of the humours, by what he calls a *fusion* (p. 92) of the blue light reflected from the choroid with the amber colour of the lens; but after all, he scarcely ventures to say more than this, that the choroid, when he looked at it, in his dissections, through the grayish retina, appeared blueish; adding, however, that during life this blueishness must be greater, from the blue colour of the venous blood with which the choroid is surcharged. To the swollen state of the choroid he attributes the propulsion of the lens through the pupil, in the last stages of glaucoma; and what is generally called the arthritic ring, seen between the sclerotica and cornea, he believes to be the effect of a dilated state of the circular venous sinus of the iris. The thinning of the sclerotica which occurs in advanced cases, he ascribes to pressure produced by the choroid.

Dr. Sichel has pointed out a symptom of glaucoma, which had escaped the notice of previous observers; namely, a change in the colour and structure of the iris. This membrane, in the part affected, assumes a grayish-slate colour, and, losing it natural fibrillary texture, becomes smooth. In this state, the iris grows thin, and is deprived of pigment, although only in patches here and there. Dr. Sichel states, that this symptom sometimes precedes the glaucomatous appearance of the humours.

In tracing the diagnosis between glaucoma and various diseases with which glaucoma might be confounded, Dr. Sichel mixes up three diseased states of the eye, which are totally different from one another, namely, fungus hæmatodes of the optic nerve, the deep-seated non-malignant deposition which so frequently follows punctured wounds of the eye, and the silvery appearance, called amaurotic cat's eye, represented by Ammon in the 10th and 11th figures of the 15th plate of the 1st volume of his *Klinische Darstellungen.*

From the remarks he makes on *opalescent amaurosis*, it is plain Dr. Sichel has never seen this curious affection. He would fain confound it with his favorite *reflet particulier du cristallin sur la choroide.*

Dr. Sichel's description of sub-choroid dropsy is good; it bears internal marks of exactness and originality, and, combined with Panizza's case,* in which the diagnosis was so mistaken, that the eye was extirpated, affords some excellent hints, of which systematic writers on eye diseases would do well to avail themselves. Dr. Sichel's notion, however, that it is only Jacob's membrane which is inflamed in sub-choroid dropsy, we cannot subscribe to, and doubt very much if that membrane is vascular in any degree.

The cases which Dr. Sichel has related (§ xvii) are well drawn up, and highly instructive. They are chiefly cases of glaucoma with arthritic ophthalmia. The first case, however, (p. 37,) appears rather to be one of dislocated lens from a blow than a pure case of glaucoma. The blow was received two months before Dr. Sichel saw the patient. Instead of immediately extracting the lens, Dr. Sichel went on endeavouring to abate the inflammation by leeches, purgatives, calomel and opium, frictions with blue ointment and laudanum, and the internal use of colchicum. Disorganizing inflammation proceeding in spite of all these remedies,

* Panizza, Sul Fungo Midollare dell' Occhio Appendice, p. 9.—Pavia, 1826.

when at last, five months after the injury, Dr. Sichel extracted the lens, no wonder that, the vitreous humour escaped, followed by profuse bleeding from the interior of the eye.

In a work of such extent, and printed in detached portions at considerable intervals of time, it might be expected, that some contradictions should occur. The following are remarkable, and deserve Dr. Sichel's attention in a new edition.

Speaking of the green colour of glaucoma, he tells us, (p. 4,) that it is sometimes " un peu sale, comme mêlé de gris ;" but at page 241, he condemns those authors who have seen any grayish tinge in the opacity of glaucoma, and gives it as his opinion, that they have confounded glaucoma with sub-choroid dropsy. At page 227, he speaks of the different shades of the yellow colour of the lens in glaucoma, and of its greater or less transparency or opacity, which is flatly in contradiction to the statement made in page 91, that the lens is entirely transparent, and normal in all respects. At page 95, he says, that neither Benedict, Rosas, nor himself had observed the fluidity of the vitreous body to be so general as Mackenzie states it to be ; but at page 123, he says it is generally fluid. At page 227, he declares that the choroid in glaucoma is inflamed in its whole elements ; but at page 241, he says glaucoma often commences with simple venous congestion of the choroid. In the same page, (p. 98,) appear two statements regarding the green colour of glaucoma totally at variance with one another. The first is, that the alteration of the choroid, the thinning and the change in colour of its pigmentous layer, joined to the yellow tinge of the crystalline, and often also of the vitreous body, alone are sufficient to explain the apparent green opacity of the bottom of the eye. The second is, that, in certain cases, the particular alteration of the choroid, without any yellow colour of the refractive media, may, by the mere refraction which the rays of light undergo in passing through these media, give rise to the green opaque appearance.

Perhaps the most defective part of Dr. Sichel's work is that which treats of the causes of glaucoma. Enslaved, as it were, by a particular notion, he does little more than tell us, that " les causes du glaucôme sont celles de la choroïdite."

As to the treatment, he declares glaucoma to be completely incurable.

> " Neither the antiphlogistic treatment," says he, " employed in all its extent and with all its rigour, nor small derivative or depletive bleedings, nor the most energetic counter-irritants, tartar-emetic ointment, the seton, cauteries or moxas to the mastoid processes and to the temples, so much vaunted by some authors, nor mineral waters, nor specific remedies extolled by others, have ever cured or even arrested the progress of a true glaucoma."

We are sorry we cannot afford more space for extracts from Dr. Sichel's interesting publication, on the general character of which we have already pronounced a favorable judgment. It grieves us to have been compelled to speak in some respects disadvantageously of the work of so assiduous an observer as we believe Dr. Sichel to be. It is an irksome task to find fault ; but the candid reader will, we think, confess, on examining Dr. Sichel's work for himself, that on this occasion the fulfilment of the task has not been unnecessary.

